# Identification of the Hub Genes and Potential Regulation Network in Chronic Hepatitis B via Bioinformatics Analysis

**DOI:** 10.1155/2022/6113807

**Published:** 2022-09-23

**Authors:** Jingjing Fan, Yong Chen, Wei Zhang, Xiaoying Zhou, Xue Bai, Caifang Chang, Yongping Han, Jinlu Liu

**Affiliations:** ^1^Department of Infectious Diseases, The First Affiliated Hospital of Hebei North University, Zhangjiakou, 075000 Hebei, China; ^2^Department of Microbiology, The First Affiliated Hospital of Hebei North University, Zhangjiakou, 075000 Hebei, China

## Abstract

**Background:**

Chronic hepatitis B (CHB) is a serious infectious disease which is induced by hepatitis B virus (HBV) infection. This project was conducted to reveal the potential mechanism in CHB development via analyzing the public clinical data.

**Methods:**

GSE33857 and GSE110217, obtained from the GEO database, were used for bioinformatics excavation. Briefly, the raw data of GSE33857 and GSE110217 were analyzed with the GEO2R, and then the expressed matrix files were generated. The matrix files was visualized as heat map with *R* software. The targets of the miRNAs were analyzed with the miRDIP database. The functional annotation and pathway enrichment were performed using “clusterProfiler” package in *R* software. The STRING database was utilized to analyze the interaction of the DEGs, and the PPI and miRNA-mRNA network were established according to the related results.

**Results:**

93 downregulated genes and 17 upregulated genes in GES33857, and 111 downregulated and 40 upregulated genes in GSE110217 were identified as the hub nodes. The targets of the DEGs in the datasets were enriched in PI3K/AKT and MAPK pathways and associated with transcriptional regulation. Moreover, PPI and miRNA-mRNA networks were also established with the DEGs and related targets in the datasets. miR-122-5p, miR-125b-5p, miR-136-5p, miR-194-5p, miR-139-5p, miR-140-5p, miR-181a-5p, and miR-29b-3p were identified as the potential biomarkers in CHB.

**Conclusion:**

Eight miRNAs, including miR-122-5p, miR-125b-5p, miR-136-5p, miR-194-5p, miR-139-5p, miR-140-5p, miR-181a-5p, and miR-29b-3p, were identified as the potential biomarkers in CHB, and the PPI and miRNA-mRNA networks were also established.

## 1. Introduction

Chronic hepatitis B (CHB) is an intractable malignant infectious disease which has high incidence in the whole world [1, 2]. At present, there are few ways to completely heal this disease. The infection of chronic hepatitis B virus is the direct factor inducing the formation and development of major patients with CHB, while some virus carriers also exhibit normal clinical indexes as well as healthy people [3]. Over the decade, increasing patients have been diagnosed with CHB year by year. HBV infections has been generally recognized as the critical reason inducing the liver-related diseases [4]. Statistically, since 1998, almost 250 million people have been confirmed to infect the CHB virus [5]. The patients with CHB may develop to various poor outcomes. At present, some antiviral drugs can impede the progression of CHB virus-induced complications, while they could not remove the persistent damage of the virus on patients in a long term [6].

Abundant research has reported that the patients with CHB generally exhibited remarkably difference in gene profile compared with healthy persons [7, 8]. miRNAs serve as critical roles in cellular activities, and the disorder of miRNA profile is also direct reason leading the formation and deterioration of the diseases [9]. For CHB, considerable reports have indicated the pathological tissues of the patients generally exhibit obvious difference in miRNA profiles [10]. With the application of HTS analysis methods, accumulating miRNAs have been investigated and successfully applied for clinical diagnosis and treatment [11]. The excavation of public data may be a promising way to reveal the potential mechanism in CHB development. Increasing researches try to illustrate the profile change in CHB development [12]. Some genes may have great potential for clinical application.

This research was conducted to analyze the potential mechanism of CHB progression via bioinformatics analysis and construct the miRNA-mRNA network to provide some reference for CHB treatment.

## 2. Materials and Methods

### 2.1. Data Source

The datasets including GSE33857 (GPL10656) and GSE110217 (GPL15018) were originated from the GEO database, and the related matrix files of the datasets were obtained via GEO2R analysis. For GSE33857, 12 normal liver tissues and 4 CHB tissues were selected as the subjects. For GSE110217, 4 normal liver tissues and 16 non-HCC CHB tissues were selected as the subjects.

### 2.2. Identification of DEGs

The matrix files of GES and GES were obtained to screen the DEGs. In brief, the genes with |logFC| ≥ 2 and *p* value <0.05 were selected as the DEGs for next analysis. The volcano map and heat map of the DEGs were figured with *R* language.

### 2.3. Enrichment Analysis

To obtain the related functional annotation and enrichment results of the DEGs, the DEGs or the related targets (for miRNAs) were analyzed using “clusterProfiler” package in *R* software [13, 14]. The related GO annotation of the DEGs was obtained for GO enrichment, and the related results of the genes in cellular component (CC), biological process (BP), and molecular function (MF) were obtained. The enrichment results were visualized with *R* language.

### 2.4. Networks Establishment and Modular Analysis

The DEGs or the related targets (for miRNAs) were analyzed with the STRING database to establish the interactions of the DEGs. Moreover, the combined score more than 0.4 was considered as statistical meaning. For modular analysis, the results obtained from STRING were analyzed with the plug-in unit of Cytoscape (MCODE). The mirDIP was utilized to predict target mRNA of miRNAs. For miRNA-mRNA network establishment, the miRNAs and the target mRNAs in GES were constructed with the Cytoscape.

## 3. Results

### 3.1. Identification of the DEGs

**Figure 1:**
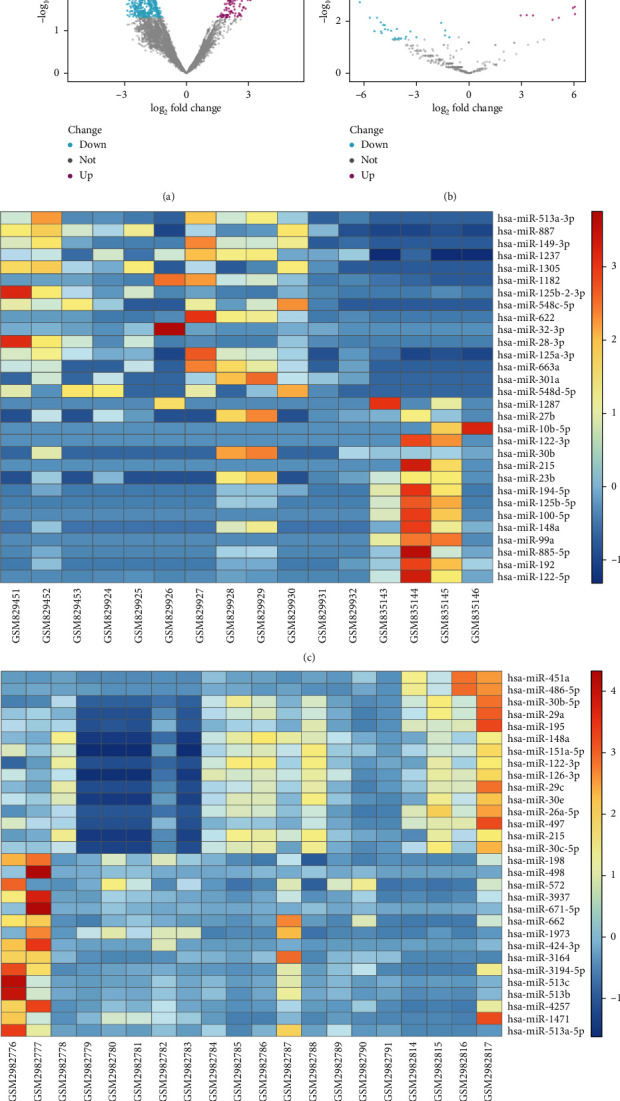
The DEGs identified from GSE33857 and GSE110217. (a, b) The hot diagrams of GSE33857 and GSE110217. (c, d) The heat maps of GSE33857 and GSE110217.

The GSE33857 and GSE110217 were analyzed with the GEO2R to identify the DEGs in the progression of CHB. In the results, 93 downregulated genes and 17 upregulated genes were found in the GES33857, and 111 downregulated and 40 upregulated genes were found in the GSE110217 (Figures 1(a) and 1(b)). The top 10 upregulated and top 10 downregulated DEGs were visualized with heat maps (Figures 1(c) and 1(d)).

### 3.2. KEGG Enrichment Analysis

**Figure 2:**
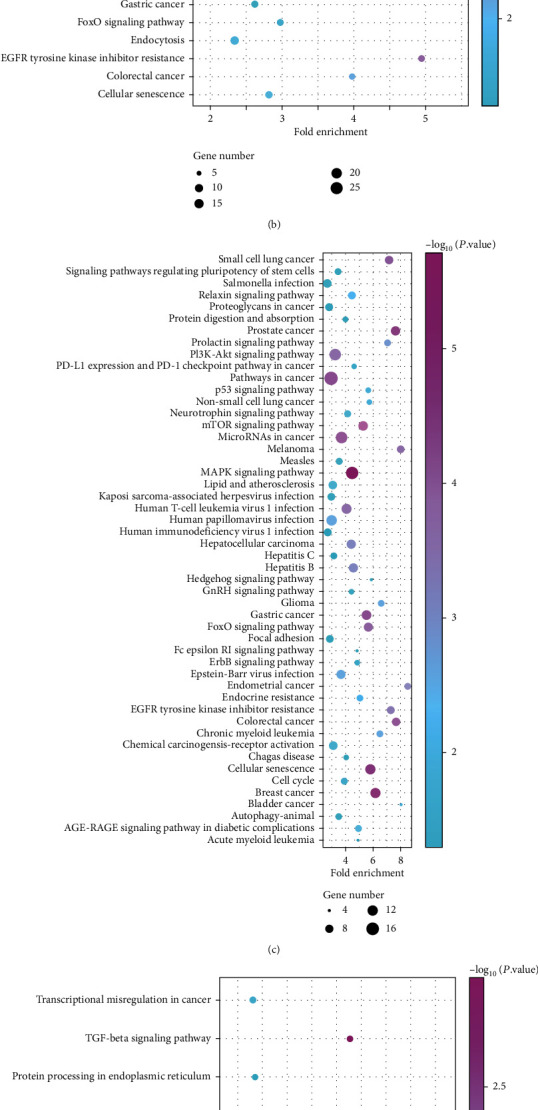
The KEGG enrichment of the DEGs in GSE33857 and GSE110217. (a) The common DEGs in GSE33857 and GSE110217. (b) The KEGG enrichment of the DEGs in GSE33857. (c) The KEGG enrichment of the DEGs in GSE110217. (d) The KEGG enrichment of common genes in GSE33857 and GSE110217.

To reveal the potential pathological mechanism of CHB, the targets of the DEGs were analyzed with the DAVID database. The results showed that 26 common genes were found in GSE33857 and GSE110217 (Figure 2(a)). The targets of the DEGs in GSE33857 were mainly enriched in PI3K/AKT and MAPK pathways, and the genes including SMAD2, FZD3, SMARCD2, PTEN, GAB1, TGFA, ARID1A, ARID2, MET, SOS2, TGFBR2, and NFE2L2 were related with the hepatocellular carcinoma related pathway (Figure 2(b)). For GSE110217, the targets of the DEGs were also enriched in PI3K/AKT and MAPK pathways, and the genes including NRAS, CCND1, AKT3, PTEN, E2F1, AXIN2, SOS2, TGFBR1, and LRP6 were enriched in hepatocellular carcinoma related pathway (Figure 2(c)). Moreover, the common targets of the DEGs in GSE33857 and GSE110217 were majorly associated with the cancer development pathways including pathways in cancer, microRNA in cancer, and transcriptional in cancer (Figure 2(d)).

### 3.3. Functional Analysis

**Figure 3:**
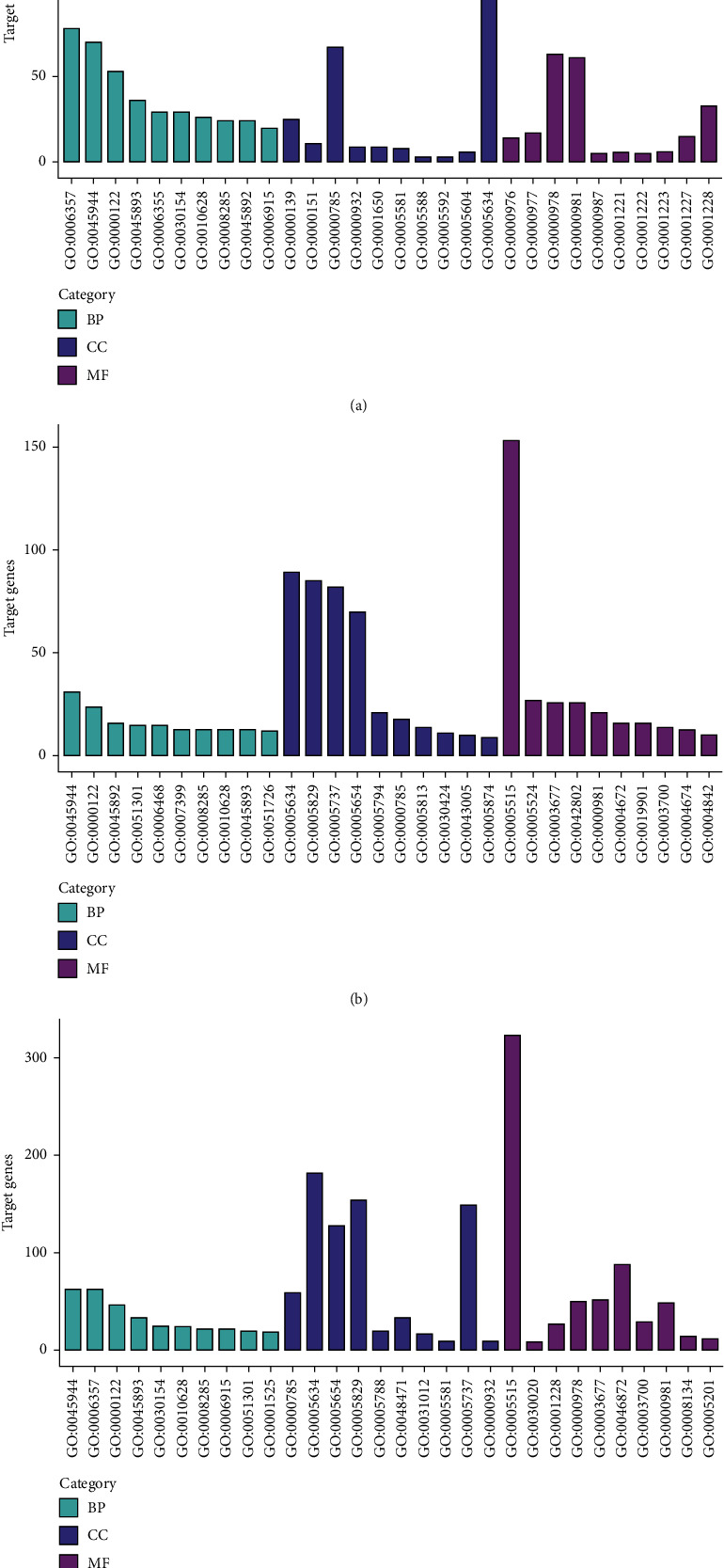
The GO enrichment of the DEGs. (a) GSE33857. (b) GSE110217. (c) The common DEGs in GSE33857 and GSE110217.

To reveal the molecular functions of the DEGs in CHB development, the targets of the DEGs in GSE33857 and GSE110217 were used for GO enrichment analysis. The results showed that the targets of the DEGs in GSE33857 and GSE110217 were located in nucleus, cytosol, nucleoplasm, and so on and were associated with the regulation of RNA transcription. Moreover, the molecular functions of these genes were related with the binding of protein, metal ion, the promoter DNA sequence, and so on (Figure 3).

### 3.4. Network Establishment

To reveal the potential molecular interaction relationship, the targets of the DEGs in GSE33857 and GSE110217 were analyzed with the STRING database. Moreover, the top 500 genes were used to establish the PPI network and miRNA-mRNA network.

**Figure 4:**
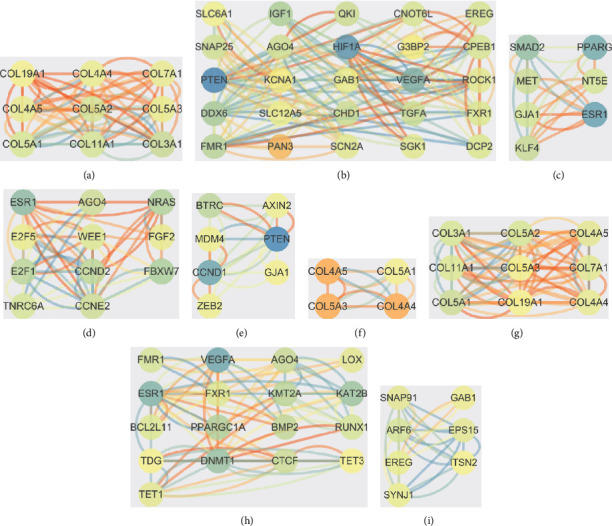
The PPI network analysis of DEGs in GSE33857 and GSE110217. (a)–(c) PPI network of the DEGs in GSE33857. (d)–(f) PPI network of the DEGs in GSE110217. (g)–(i) PPI network of the DEGs in common genes.

**Figure 5:**
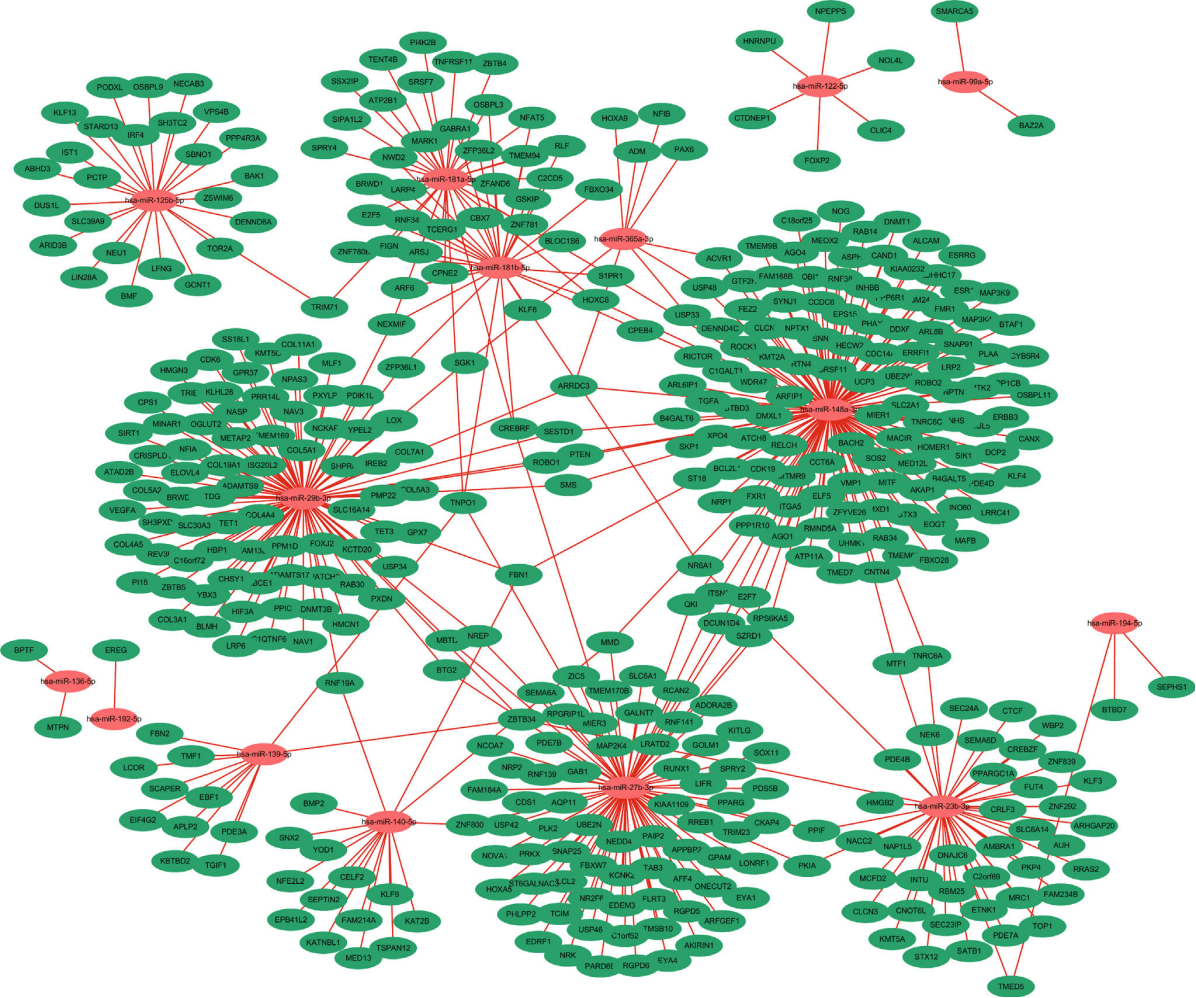
The miRNA-mRNA network of the common genes in GSE33857 and GSE110217.

The results showed that 3 clusters were screened in both GSE33857 and GSE110217 via MCODE plug-in unit. For GSE33857, the targets of DEGs including PTEN, HIF1, VEGFA, SMAD2, PPARG, and ESR1 were identified as the hub nodes (Figures 4(a)–4(c)). ESR1, FBXW7, NRAS, PTEN, CCND1, and BTRC were identified as the hub nodes in GSE110217 (Figures 4(d)–4(f)). For common targets VEGFA, ESR1, DNMT1, KMT2A, and KAT2B were identified as hub nodes (Figures 4(g)–4(i)). Moreover, the miRNA-mRNA network was also established based on the targets of common DEGs in GSE33857 and GSE110217, and the results showed that 15 miRNA nodes and 351 mRNA nodes were included in the network (Figure 5).

## 4. Discussion

Chronic hepatitis B (CHB) is still a malignant infectious disease which seriously threatens the health of human and provides great challenge for the modern medical system [15]. In this research, the bioinformatics analysis was applied to investigate the potential regulation mechanism in the progression of CHB, and the GSE33857 and GSE110217 were used to investigate the potential biomarkers.

miRNAs have been confirmed as critical roles in regulating the normal progression of cells, and miRNA dysfunction has been proved as a direct reason leading the malignant progression of multiple diseases [16]. This investigation also discovered that the miRNA profile in the patient's tissues exhibited significant difference compared with that in the normal subjects [17]. 15 miRNAs were selected as the hub nodes in CHB. The research has confirmed that reduced miR-122-5p is closely related with the progression of HBV-induced liver fibrosis, and miR-122-5p can effectively impeded the deterioration of the symptom via targeting IL7R [18]. Increased miR-125b-5p has been identified as a serum biomarker for HBV infection by several reporters [19]. Deng et al. has also confirmed that miR-125b-5p can interact with LIN28B to induce the posttranscriptional regulation of HBV [20]. He et al. have indicated that decreased miR-136-5p can aggravate the liver cancer development induced by HBV [21]. miR-194-5p is also upregulated in the HBV host cells [22]. Moreover, miR-139-5p has been found to block the malignant behaviors of hepatocellular carcinoma cells [23]. miR-140-5p has also been confirmed to impede the EMT process via targeting TGFBR1 [24]. miR-181a-5p inhibited the cellular malignant proliferation via repressing EGR1 in hepatocellular carcinoma [25]. Zhou et al. have indicated that miR-29b-3p can impede the metastasis of hepatocellular carcinoma via blocking DNMT3A [26].

miRNA is characterized with repressing the translation via binding with the 3′-UTR of the proteins. In this research, the targets of the miRNAs have been screened by miRDIP targets. PTEN, HIF1, VEGFA, SMAD2, PPARG, and ESR1 were identified as the hub nodes in GSE3857. ESR1, FBXW7, NRAS, PTEN, CCND1, and BTRC were identified as the hub nodes in GSE110217. VEGFA, ESR1, DNMT1, KMT2A, and KAT2B were identified as hub nodes in common targets of GSE3857 and GSE110217. DNMT1 has been identified as a serum biomarker event in cancer development, and DNMT1 has been also confirmed to be associated with HBV-related liver cancer [27]. In this research, DNMT1 was identified as the target of miR-148a-3p. FBXW7 plays a tumor supporter role in multiple tumors, and increased FBXW7 has been confirmed to be closely related with resistance of liver cancer cells on sorafenib [28]. This research found that FBXW7 served as a target of miR-27b-3p, and miR-27b-3p was dramatically reduced in HBV-infected tissues. Neovascularization is a biomarker event in the progression of some tumors. For gaining more nutrient, the tumor focuses generally exhibit aberrant angiopoiesis. VEGFA serves as critical role in driving the process of angiopoiesis in local low oxygen environment, which has been also identified as the biomarker in cancer development. Increased VEGFA is associated with liver cancer development, and several reports have indicated that VEGFA inhibition can extremely block the malignant behaviors of the cancer cells [29]. Moreover, increased VEGFA has been observed in HBV-related liver cancer.

Abundant reports have confirmed that HBV infection can induce the aberrant activities of the pathways in liver cells. This research has indicated that the DEGs in GSE33857 and GSE110217 were enriched in PI3K/AKT and MAPK pathways. PI3K/Akt signaling pathway is an important signal transduction pathway in cells, which plays an important role in inhibiting apoptosis and promoting cell survival and proliferation. Akt is an important downstream target kinase in PI3K signal transduction pathway, which has silk threonine kinase activity. When cells are stimulated by extracellular signals, PI3K activation generates PIP3 to translocate Akt to the cell membrane and localize near PDK1 and PDK2. The conformation of Akt changes, exposing its phosphorylation site. Subsequently, Akt activation further acts on its downstream molecules, thus participating in cell growth, development, differentiation, and proliferation. The abnormal activation of PI3K/AKT pathways is related with the aberrant proliferation and enhanced invasive ability of tumor cells. HBV has been confirmed to induce the antiapoptosis ability of liver cancer cells via activating the PI3K/AKT pathway [29]. Moreover, MAPK pathway dysfunction could drive the deterioration of HBV-mediated liver cancer via promoting the transcription of surface protein hPIAS1 of HBV [30].

In conclusion, eight miRNAs, including miR-122-5p, miR-125b-5p, miR-136-5p, miR-194-5p, miR-139-5p, miR-140-5p, miR-181a-5p, and miR-29b-3p, were identified as the potential biomarkers in CHB, and the PPI and miRNA-mRNA networks were also established. The limitations of this study lie in the use of too few data sets and the lack of experimental validation. The next study should further verify the effect of these candidate biomarkers on CHB in vivo and in vitro.

## Data Availability

Data to support the findings of this study is available on reasonable request from the corresponding author.
